# Supplementary material for: Insights into the recognition mechanism in the UBR box of UBR4 for its specific substrates
Source: Commun Biol. 2023 Nov 29;6:1214. doi: 10.1038/s42003-023-05602-7 (PMC10687169; doi:10.1038/s42003-023-05602-7)
Supplement: Supplementary file 2 — Supplementary information [file 42003_2023_5602_MOESM2_ESM.pdf]

Supplementary Figure 1.

a

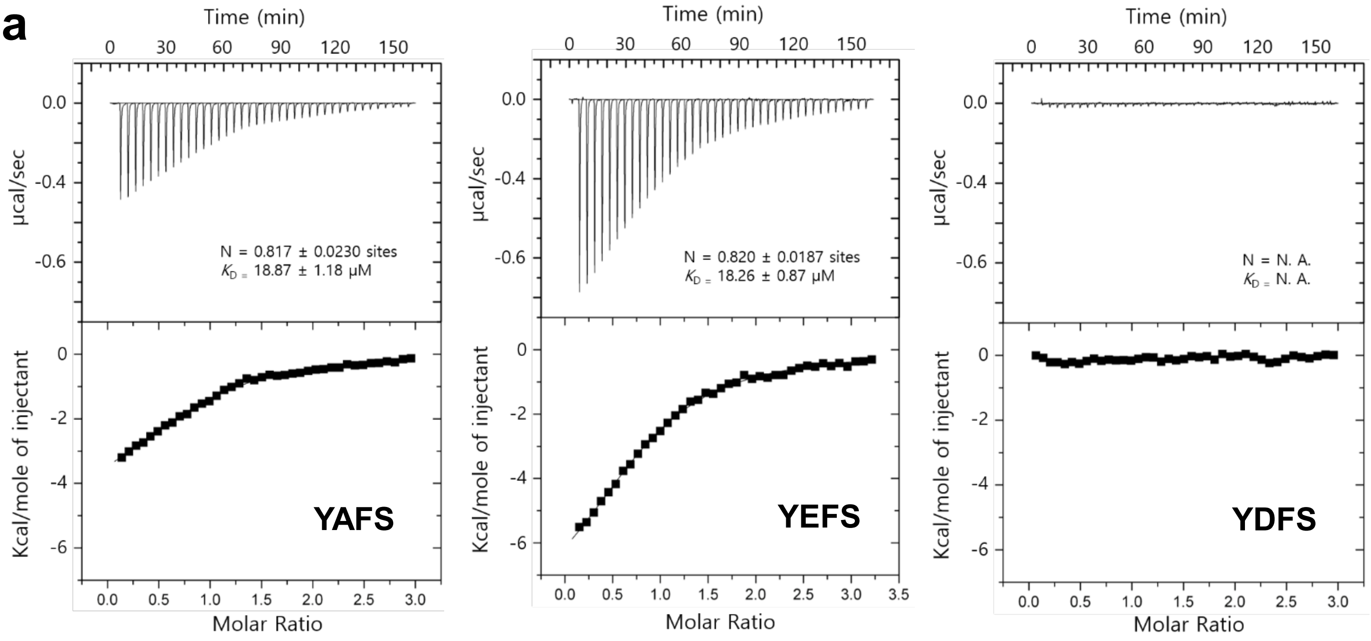

b

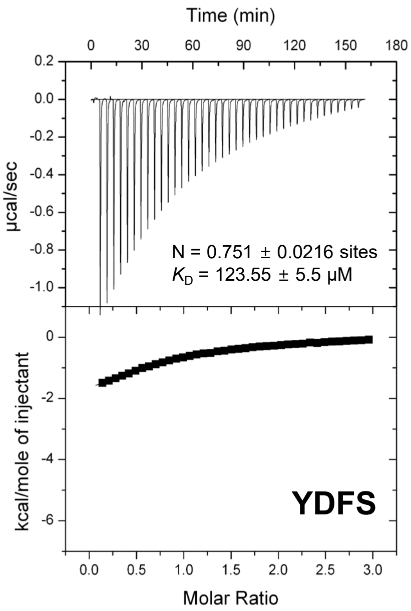

Peptide	K_D (uM)
YDFS	124 ± 6

* Limit of detection : 225uM

Supplementary Fig. 1. Raw isothermal titration calorimetry data for $UBR4^{UBR}$ and three ligands of the YXFS. (**a**) YAFS, YEFS, and YDFS. The concentrations of $UBR4^{UBR}$ protein is 0.05 mM and peptides are 1 mM. (**b**) YDFS that is 4.5 times higher in concentrations. The concentrations of $UBR4^{UBR}$ protein is 0.225 mM and YDFS peptide is 4.5 mM.

Supplementary Figure 2.

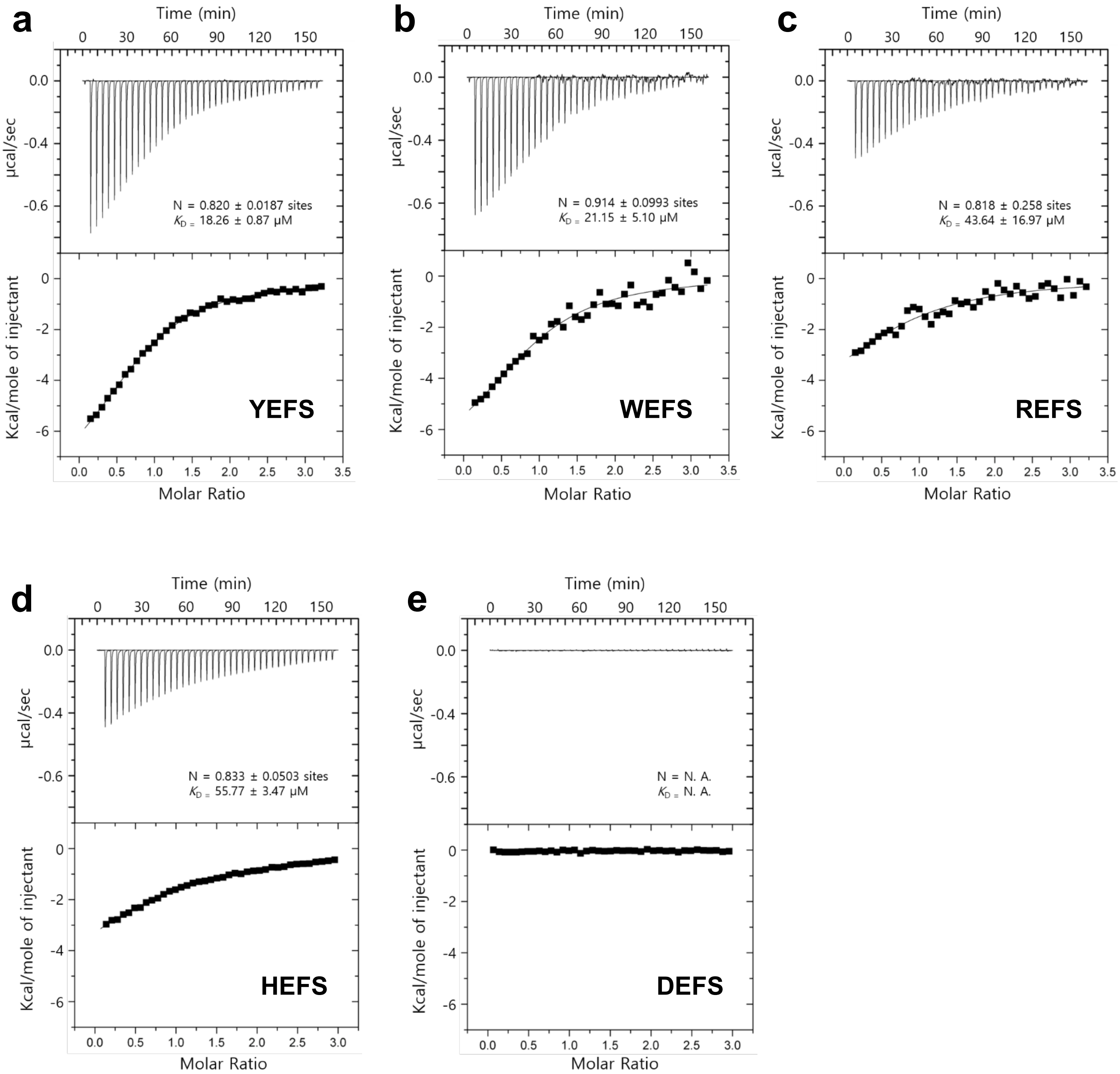

Supplementary Fig. 2. Raw isothermal titration calorimetry data for $UBR4^{UBR}$ and five ligands of the XEFS. The concentrations of UBR4UBR protein is 0.05 mM and peptides are 1 mM. (**a**) Type-2 N-degron with aromatic ring, YEFS. (**b**) Type-2 N-degron with aromatic ring, WEFS. (**c**) Type-1 N-degron, REFS. (**d**) Type-1 N-degron with aromatic ring, HEFS. (**e**) Negative control, DEFS

Supplementary Figure 3.

Thermal shift assay melting curve **(YXFS)**

Control
2mM (YXFS)
1mM (YXFS)

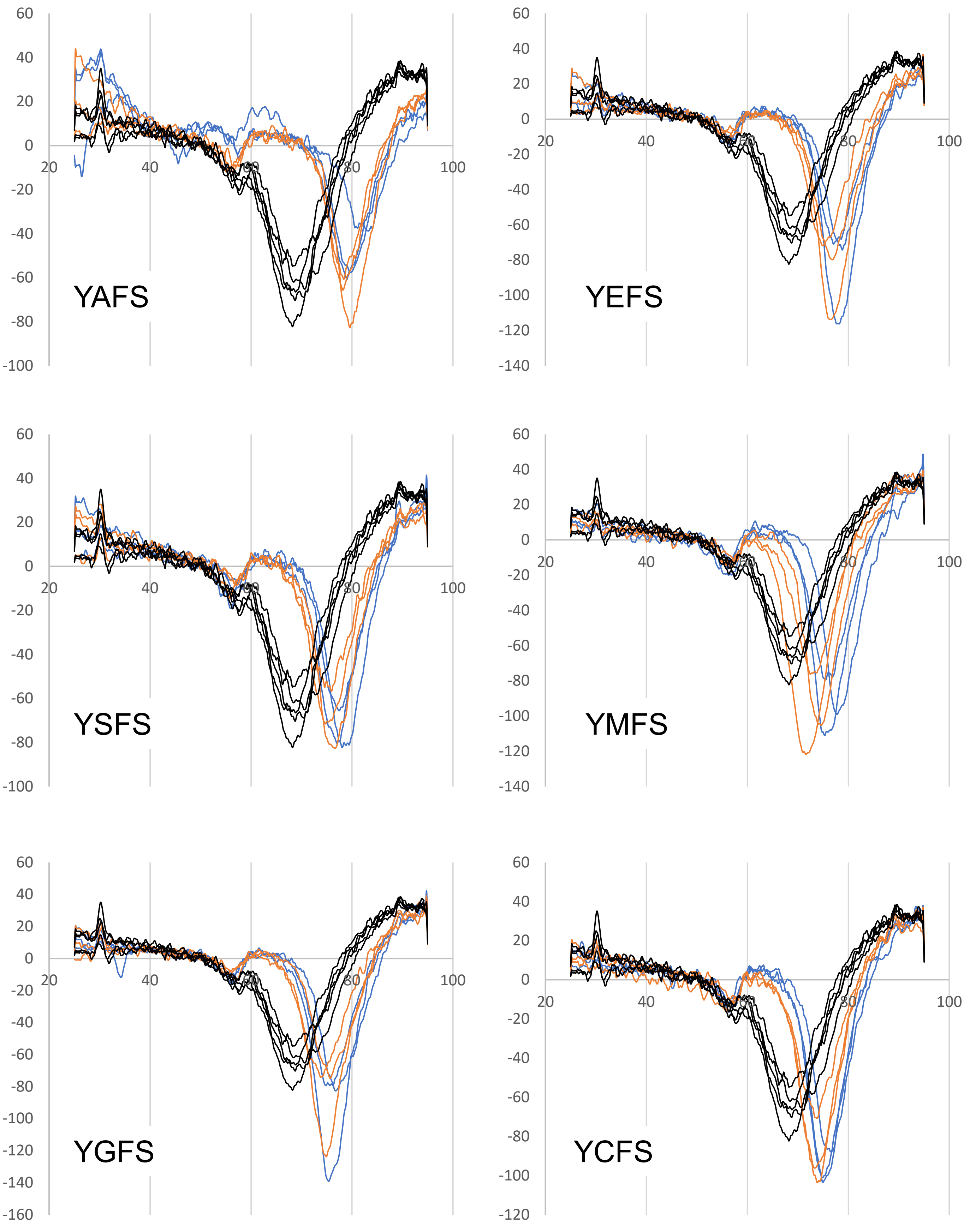

Supplementary Figure 3.

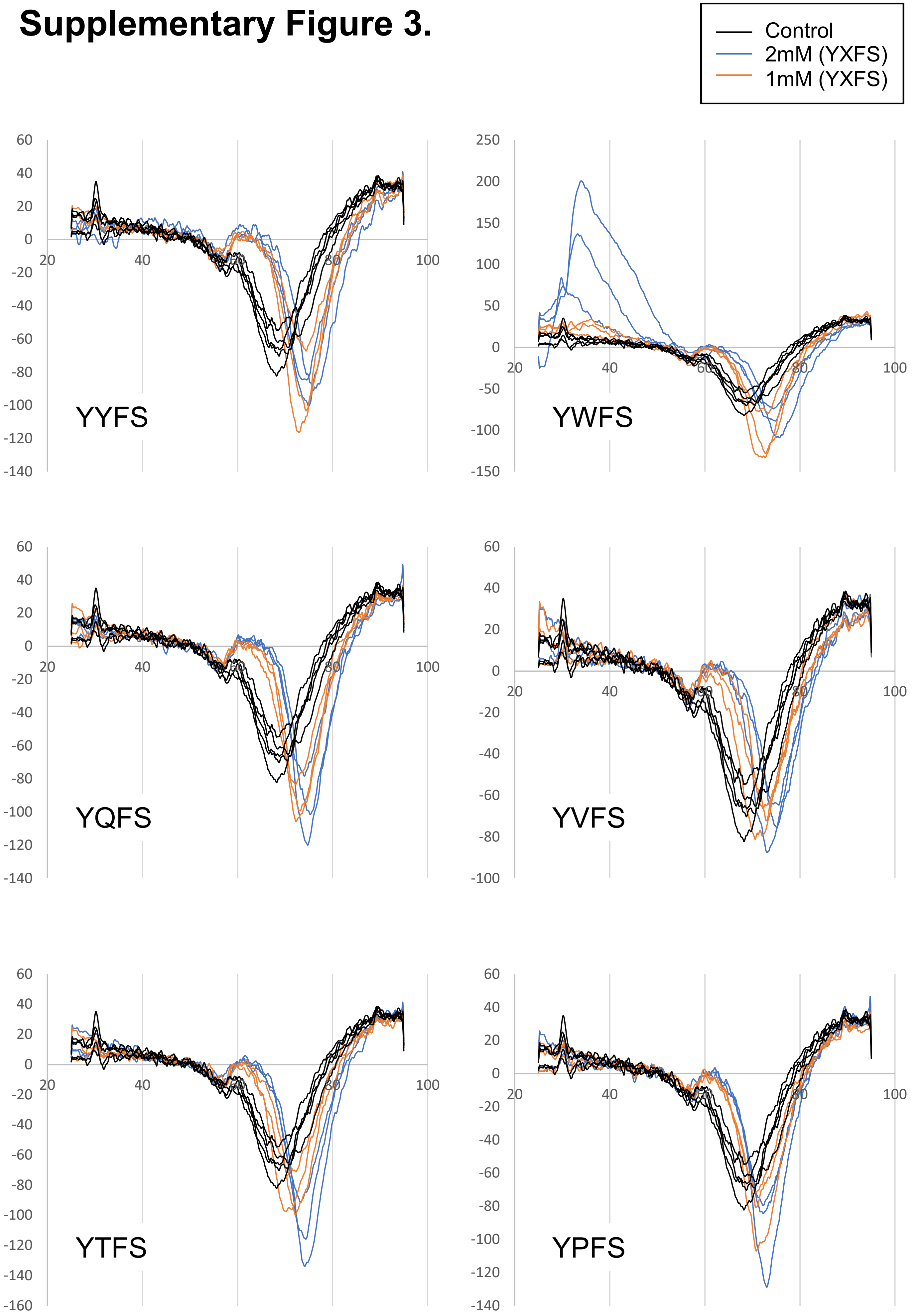

Supplementary Figure 3.

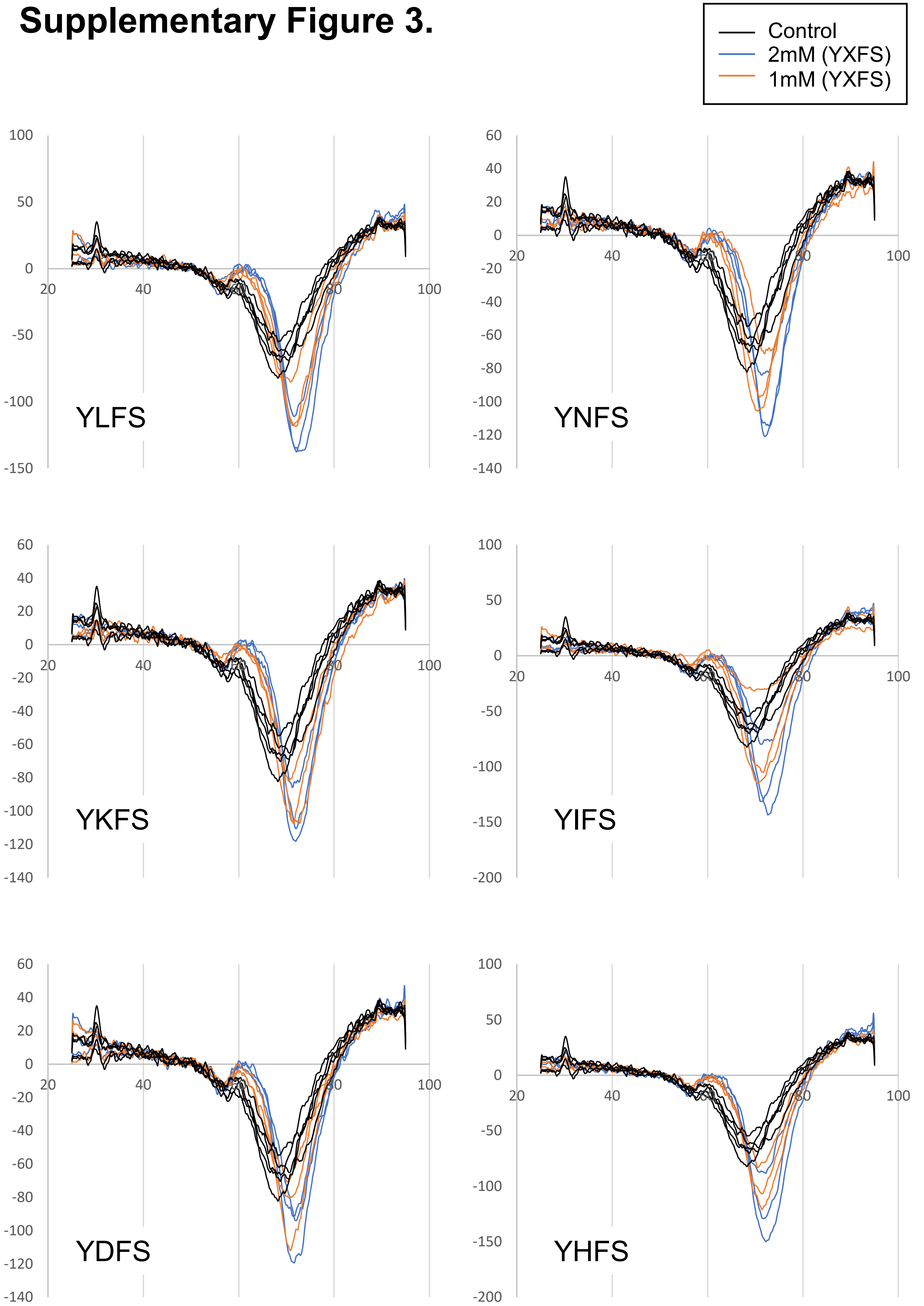

Supplementary Figure 3.

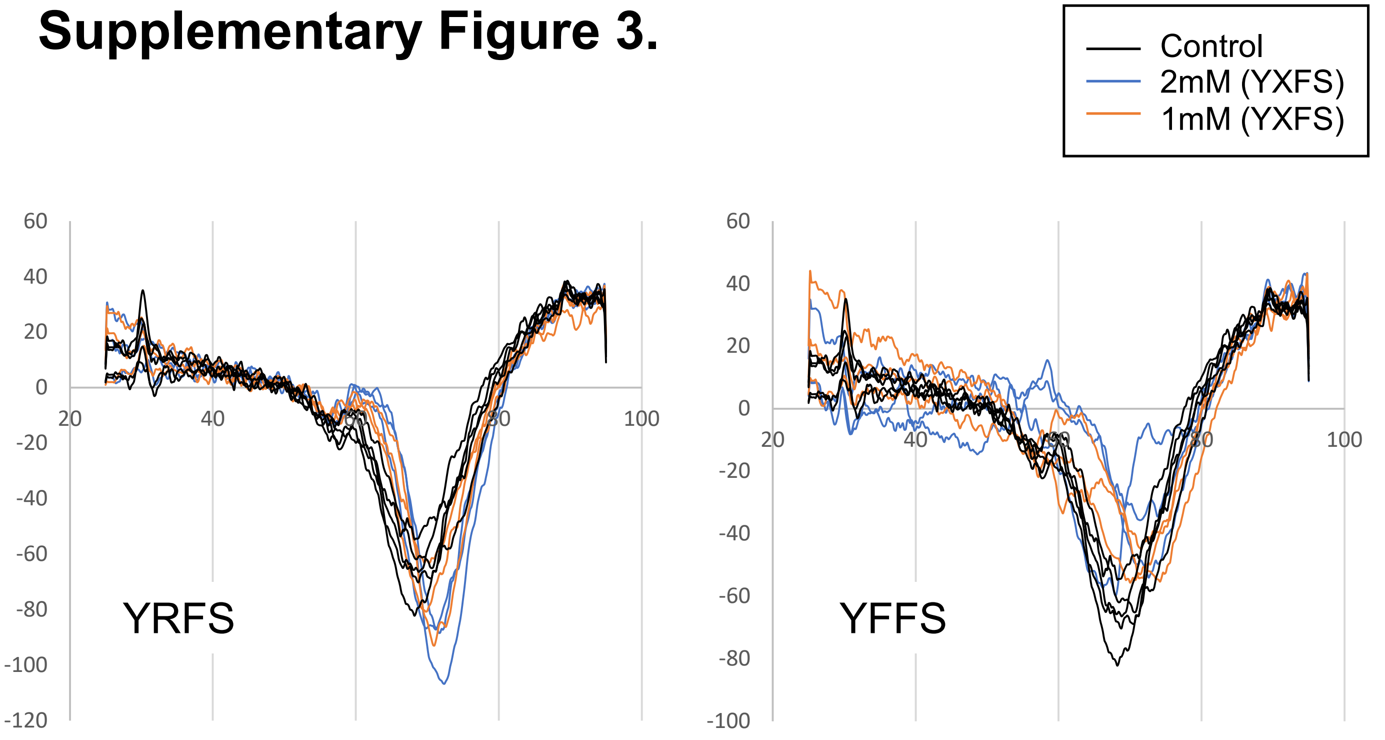

Supplementary Figure 3.

YXFS 2 mM		
	Average Tm (°C)	ΔTm (°C)
Control	68.64 ± 1.36	-
YAFS	80.67 ± 2.23	12.06 ± 2.37
YEFS	77.87 ± 0.90	9.26 ± 1.20
YSFS	77.47 ±0.50	8.86 ± 0.94
YMFS	76.27 ± 1.33	7.66 ± 1.55
YGFS	76.13 ± 0.70	7.52 ± 1.06
YCFS	75.47 ±0.99	6.86 ± 1.26
YYFS	75.20 ± 0.72	6.59 ± 1.07
YWFS	74.93 ± 0.61	6.32 ± 1.00
YQFS	74.73 ± 0.70	6.12 ± 1.06
YVFS	74.53 ± 1.36	5.92 ± 1.57
YTFS	73.93 ± 0.64	5.32 ± 1.02
YPFS	72.47 ± 0.46	3.86 ± 0.92
YLFS	72.13 ± 0.50	3.52 ± 0.94
YNFS	72.07 ± 0.70	3.46 ± 1.06
YKFS	71.73 ± 0.46	3.46 ± 0.70
YIFS	71.67 ± 0.81	3.06 ± 1.13
YDFS	71.67 ± 0.31	3.06 ± 0.85
YHFS	71.60 ± 0.72	2.99 ± 1.07
YRFS	71.33 ± 1.36	2.72 ± 1.57
YFFS	70.67 ± 2.39	2.06 ± 2.51

YXFS 1 mM		
	Average Tm (°C)	ΔTm (°C)
Control	68.64 ± 1.36	-
YAFS	78.73 ± 0.76	10.12 ± 1.10
YEFS	76.13 ± 0.99	7.52 ± 1.26
YSFS	75.80 ± 0.92	7.19 ± 1.21
YMFS	72.87 ± 1.55	4.26 ± 1.74
YGFS	74.73 ± 0.90	6.12 ± 1.20
YCFS	73.67 ± 0.23	5.06 ± 0.82
YYFS	73.93 ± 0.81	5.32 ± 1.13
YWFS	72.93 ± 0.42	4.32 ± 0.89
YQFS	72.60 ± 0.53	3.99 ± 0.95
YVFS	71.93 ± 1.22	3.32 ± 1.46
YTFS	71.00 ± 1.04	2.39 ± 1.31
YPFS	70.73 ± 0.12	2.12 ± 0.80
YLFS	71.53 ± 0.50	2.92 ± 0.94
YNFS	71.13 ± 0.76	2.52 ± 1.10
YKFS	71.07 ± 0.61	2.46 ± 1.00
YIFS	70.67 ± 0.90	2.06 ± 1.20
YDFS	70.53 ± 0.31	1.92 ± 0.85
YHFS	71.13 ± 0.46	2.52 ± 0.92
YRFS	70.13 ± 0.76	1.52 ± 1.10
YFFS	71.93 ± 2.12	3.32 ± 2.26

Supplementary Fig. 3. The melting curve of TSA on YXFS with UBR4^{UBR}. The highest concentration (2 mM) of melting curve shown as blue color and the second highest concentration (1 mM) of melting curve shown in orange. Each result has shown with the melting curve of control as black color.

Supplementary Figure 4.

Thermal shift assay melting curve **(XEFS)**

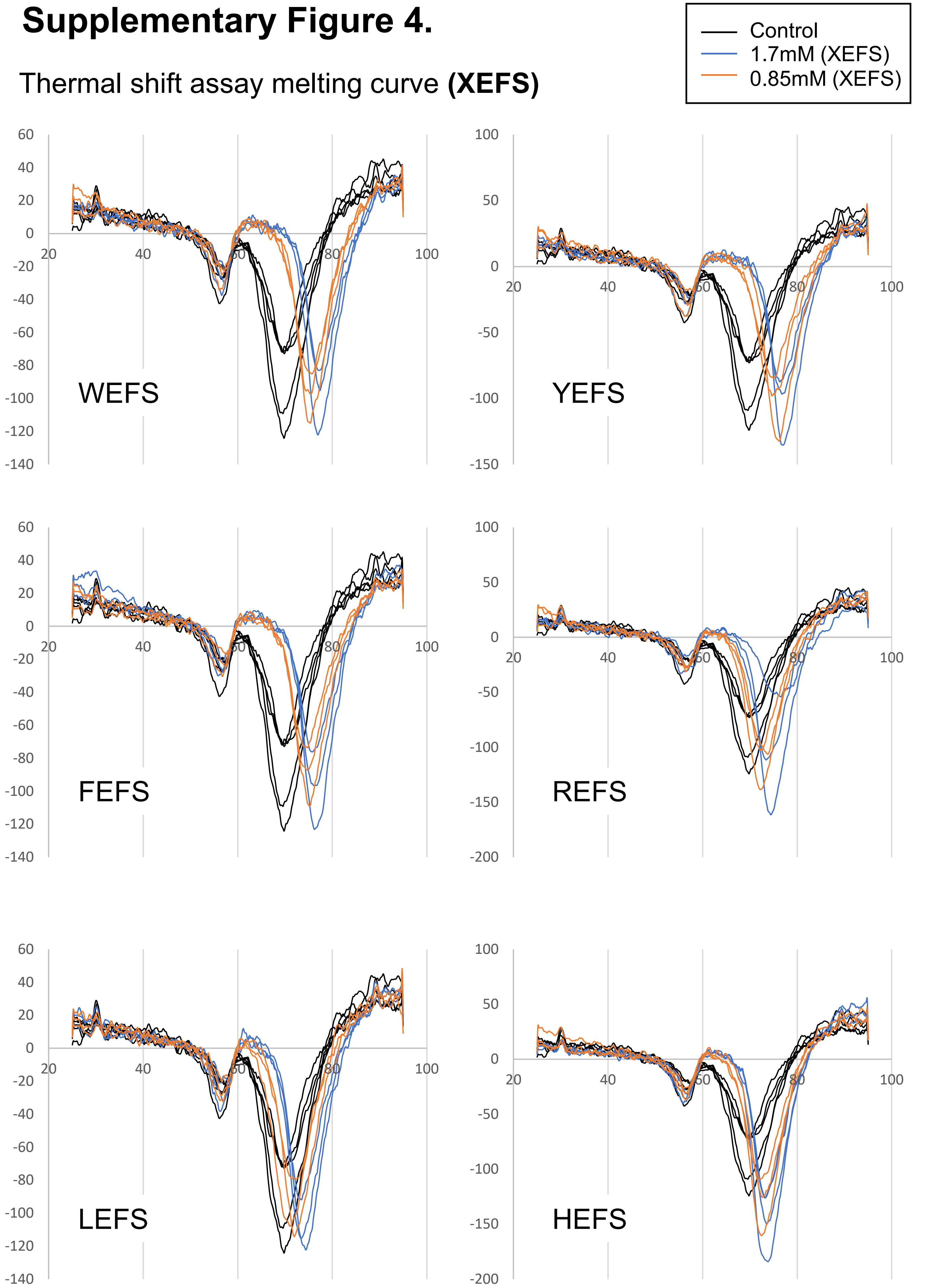

Supplementary Figure 4.

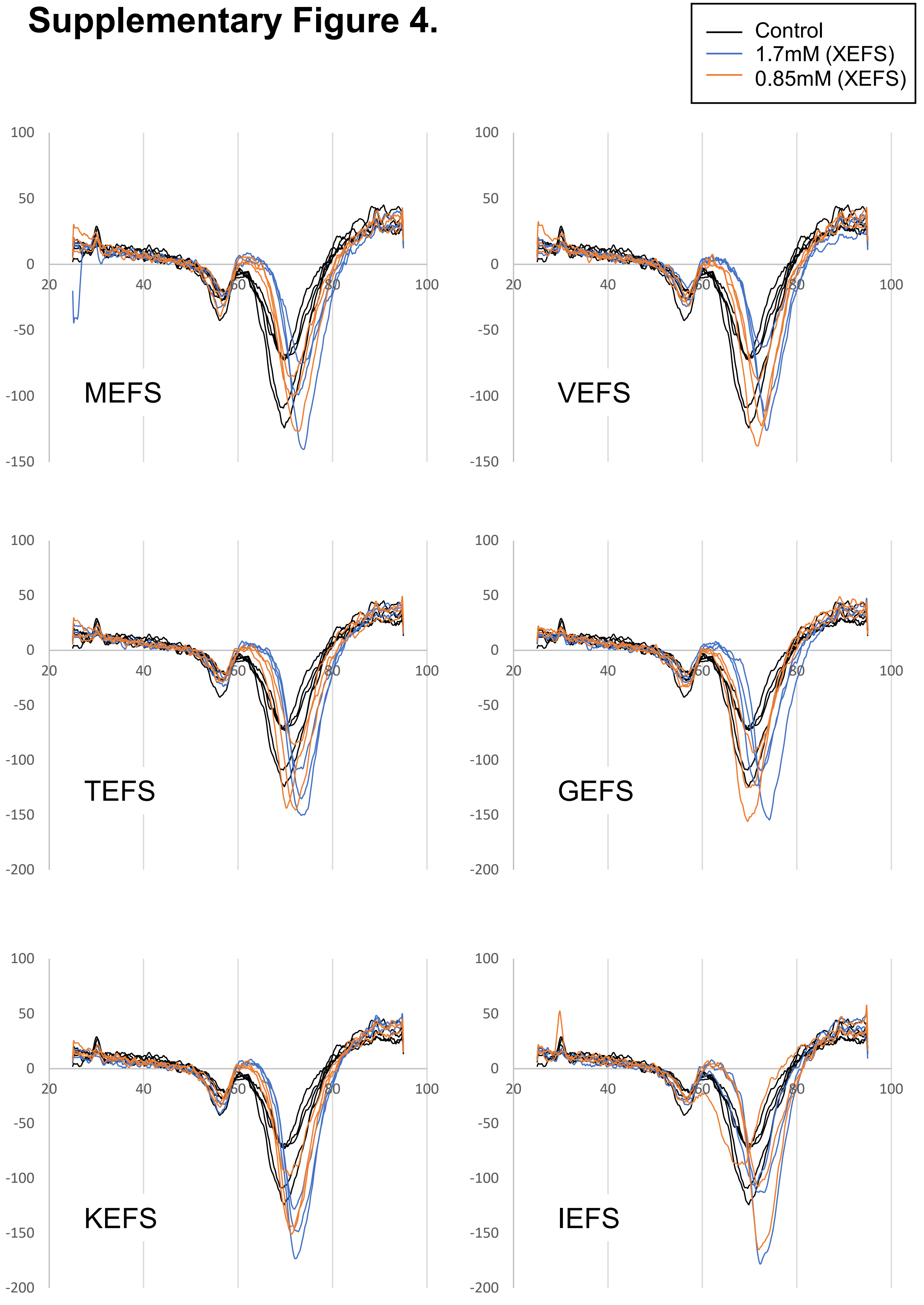

Supplementary Figure 4.

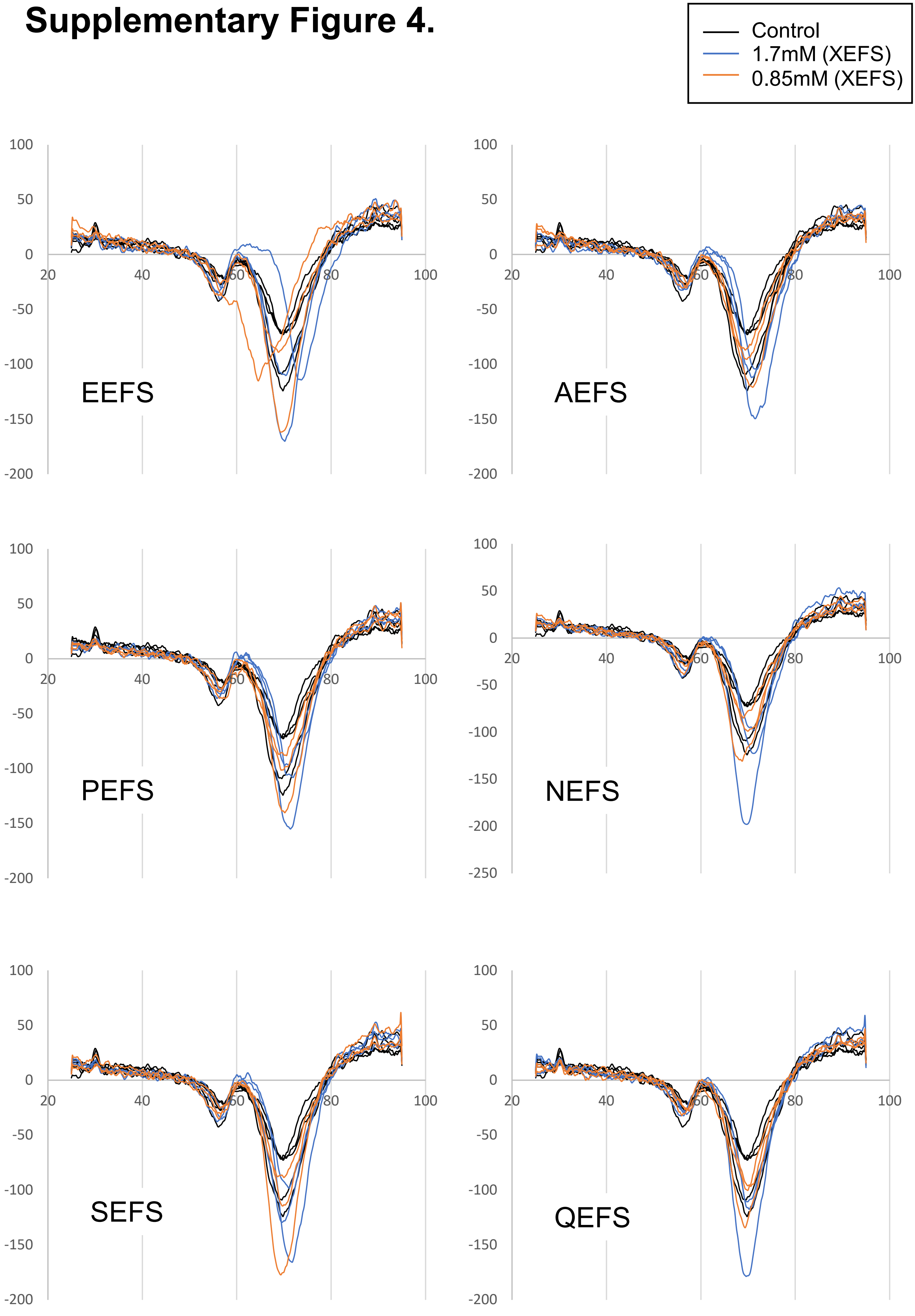

Supplementary Figure 4.

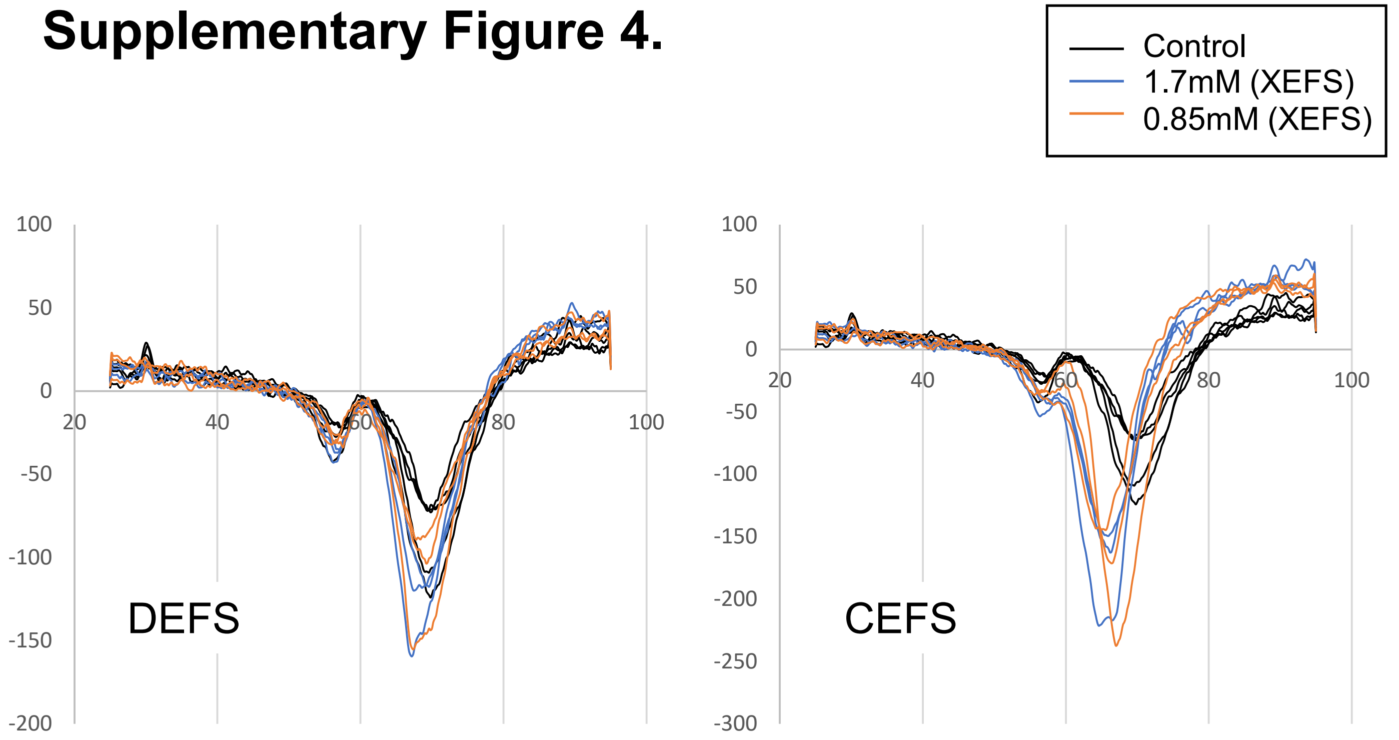

Supplementary Figure 4.

XEFS 1.7 mM		
	Average Tm (°C)	ΔTm (°C)
Control	69.76 ± 0.91	-
WEFS	77.20 ± 0.20	7.44 ± 0.93
YEFS	76.73 ± 0.31	6.97 ± 0.96
FEFS	76.07 ± 0.23	6.31 ± 0.94
REFS	74.80 ± 1.64	5.04 ± 1.87
LEFS	73.73 ± 0.58	3.97 ± 1.08
HEFS	73.53 ± 0.31	3.77 ± 0.96
MEFS	73.30 ± 0.50	3.57 ± 1.04
VEFS	73.27 ± 0.31	3.51 ± 0.96
TEFS	73.07 ± 0.58	3.31 ± 1.08
GEFS	72.73 ± 1.33	2.97 ± 1.61
KEFS	72.33 ± 0.42	2.57 ± 1.00
IEFS	72.07 ± 1.01	2.31 ± 1.36
EEFS	71.40 ± 1.91	1.64 ± 2.11
AEFS	71.27 ± 0.42	1.51 ± 1.00
PEFS	71.20 ± 0.92	1.44 ± 1.29
NEFS	70.80 ± 0.87	1.04 ± 1.26
SEFS	70.80 ± 1.11	1.04 ± 1.44
QEFS	69.87 ± 0.31	0.11 ± 0.96
DEFS	68.13 ± 1.29	-1.63 ± 1.58
CEFS	65.60 ± 0.87	-4.16 ± 1.26

XEFS 0.85 mM		
	Average Tm (°C)	ΔTm (°C)
Control	69.76 ± 0.91	-
WEFS	75.47 ± 0.12	5.71 ± 0.92
YEFS	75.13 ± 0.61	5.37 ± 1.10
FEFS	76.93 ± 0.23	5.17 ± 0.94
REFS	72.73 ± 0.92	2.97 ± 1.30
LEFS	71.73 ± 0.64	1.97 ± 1.12
HEFS	72.40 ± 0.20	2.64 ± 0.93
MEFS	71.80 ± 0.92	2.04 ± 1.29
VEFS	71.93 ± 0.42	2.17 ± 1.00
TEFS	71.47 ± 1.10	1.71 ± 1.43
GEFS	70.00 ± 0.69	0.24 ± 1.15
KEFS	71.47 ± 0.50	1.71 ± 1.04
IEFS	70.87 ± 1.79	1.11 ± 2.01
EEFS	67.60 ± 2.62	-2.16 ± 2.77
AEFS	70.07 ± 0.83	0.31 ± 1.24
PEFS	69.93 ± 0.46	0.17 ± 1.02
NEFS	69.27 ± 0.64	-0.49 ± 1.12
SEFS	69.73 ± 0.31	-0.03 ± 0.96
QEFS	69.73 ± 0.46	-0.03 ± 1.02
DEFS	68.33 ± 0.90	-1.43 ± 1.28
CEFS	66.33 ± 0.70	-3.43 ± 1.15

Supplementary Fig. 4. The melting curve of TSA on XEFS with $UBR4^{UBR}$. The highest concentration (1.7 mM) of melting curve (blue) and the second highest concentration (0.85 mM) of melting curve (orange) shown in different color. Each result has shown with the melting curve of control as black color.

Supplementary Figure 5.

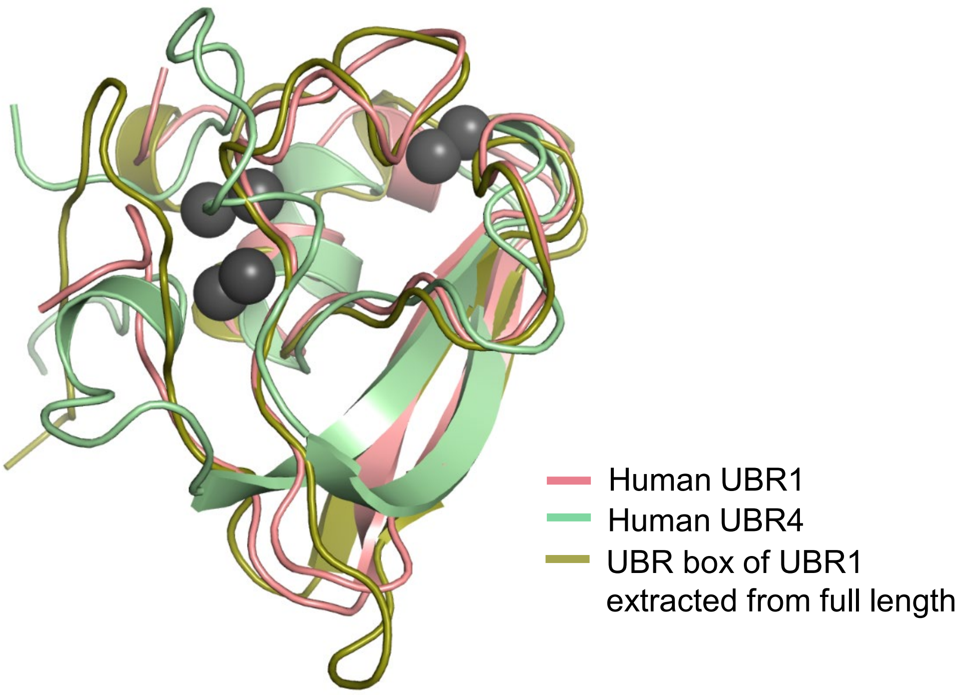

Protein 1	Protein 2	RMSD value
Human UBR1	UBR box of UBR1 extracted from full length	1.521 (51 to 51 atoms)
Human UBR1	Human UBR4	2.227 (43 to 43 atoms)
UBR box of UBR1 extracted from full length	Human UBR4	4.666 (40 to 40 atoms)

Supplementary Fig. 5. The crystal structure of the UBR box of UBR1 extracted from full length UBR1 (PDB ID : 7MEX) aligned with Human UBR boxes of UBR1 and UBR4. The UBR box of UBR1 extracted from full length structure showed high similarity with Human UBR box of UBR1. Human UBR1, UBR box of UBR1 extracted from full length, and Human UBR4 represent salmon, deepolive, and palegreen colors, respectively.

Supplementary Figure 6.

a The LC3B-fusion system

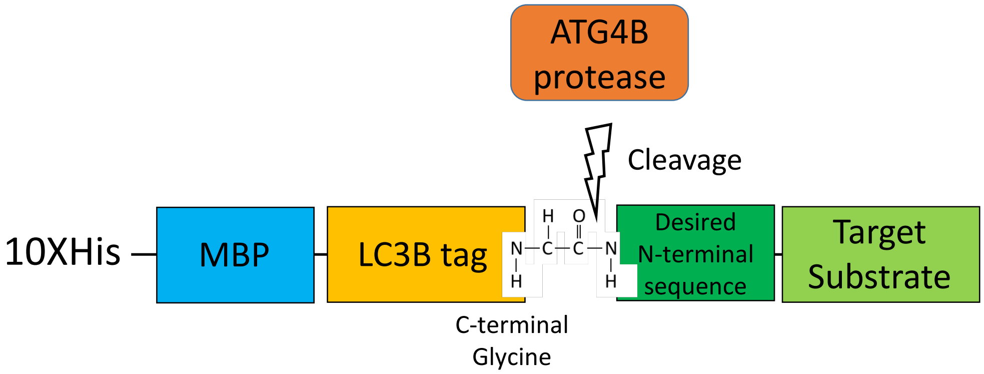

b

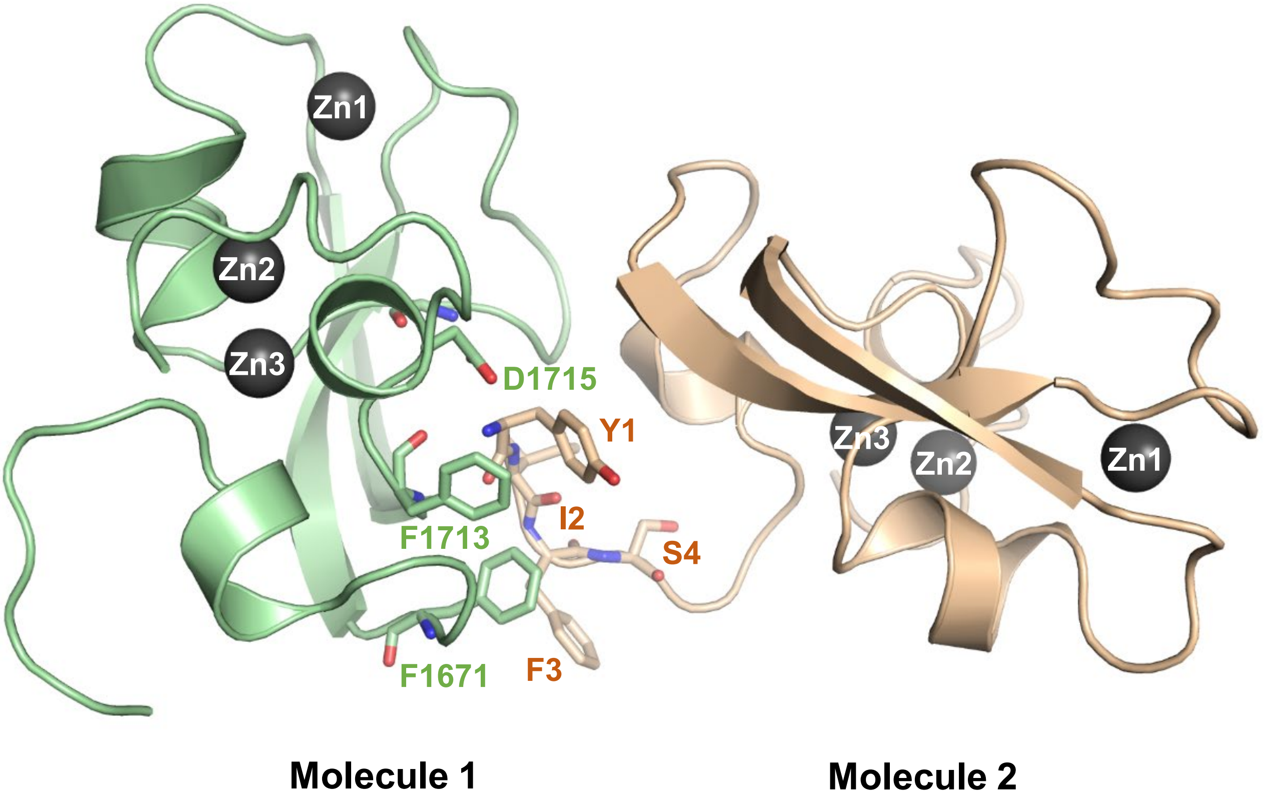

c

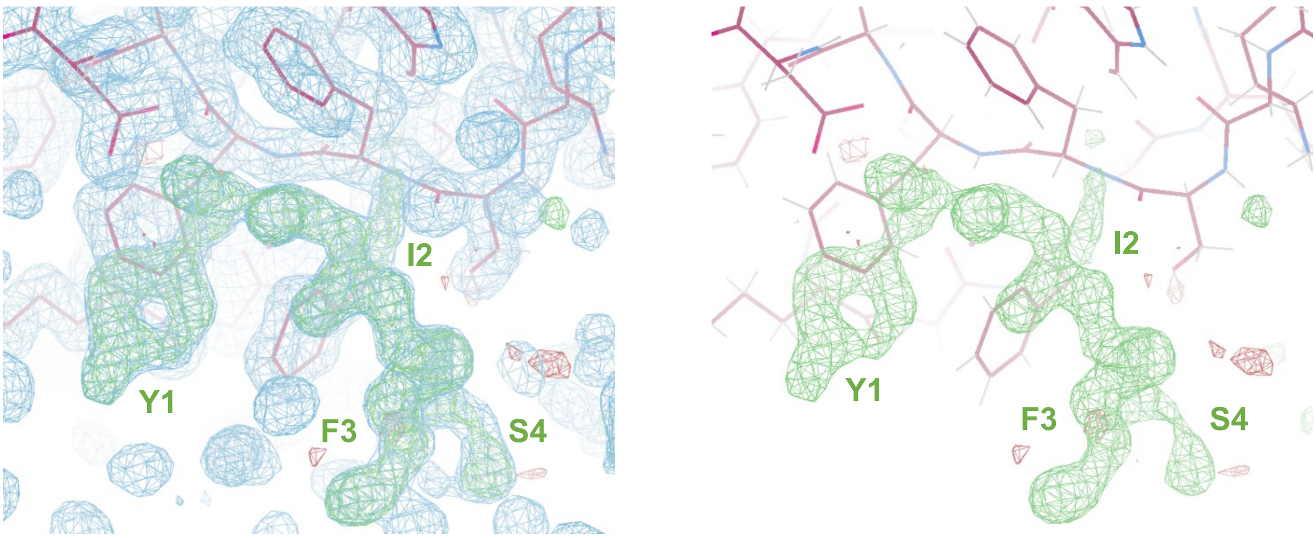

Supplementary Figure 6.

Supplementary Fig. 6. (**a**) Domain structure of the LC3B-fusion system. ATG4B protease cleavage at the C-terminal glycine of LC3B tag. (**b**) The crystal structure of two molecules of YIFS-UBR4^{UBR}. The N-terminus amino acid of molecule 2 (colored as wheat) is bound to the ligand-binding pocket of molecule 1 (colored as palegreen) through crystal packing. In this structure, two phenylalanines in molecule 1 interact with N-terminal tyrosine residue of molecule 2. (**c**) Density of an omit map of the bound peptide (YIFS, colored as green).

Supplementary Figure 7.

a

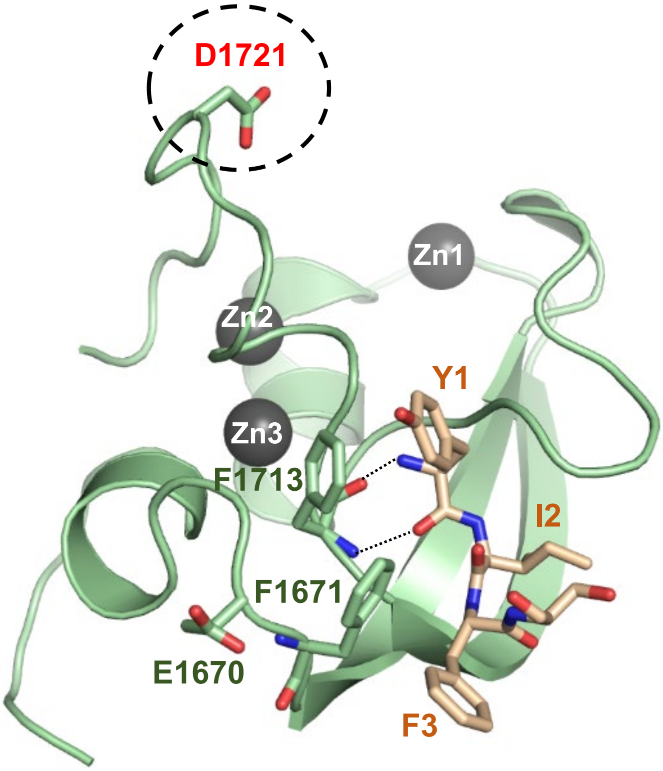

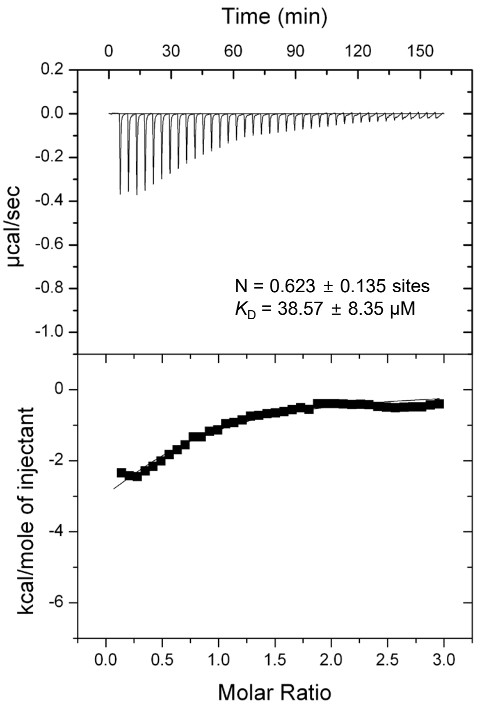

UBR4^{UBR} (D1721A) with YEFS

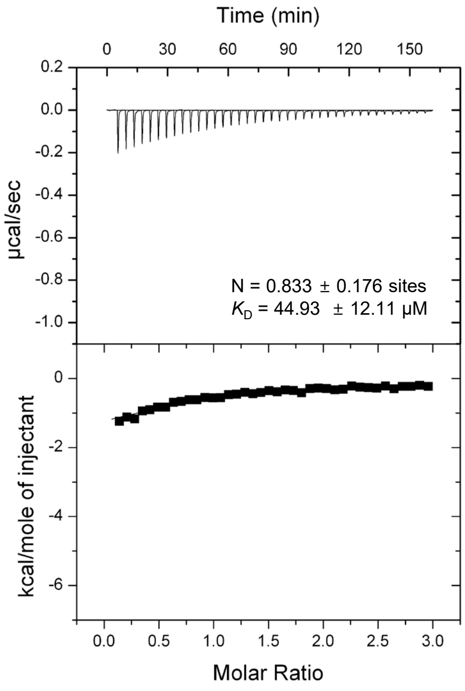

UBR4^{UBR} (D1721A) with REFS

Supplementary Figure 7.

b

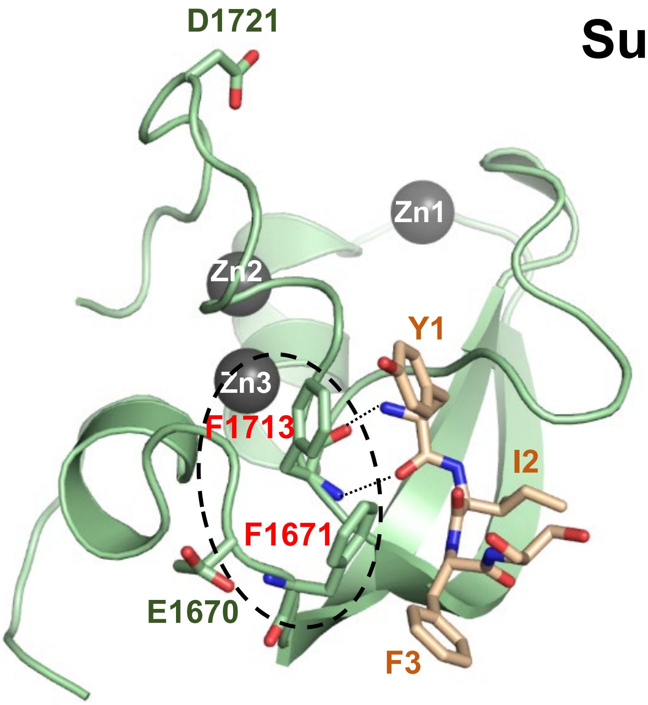

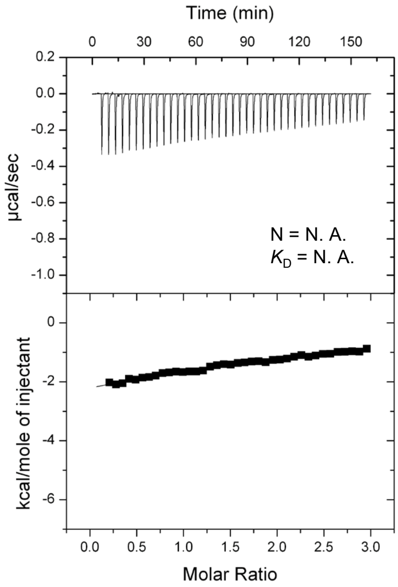

UBR4^{UBR} (F1671A) with YEFS

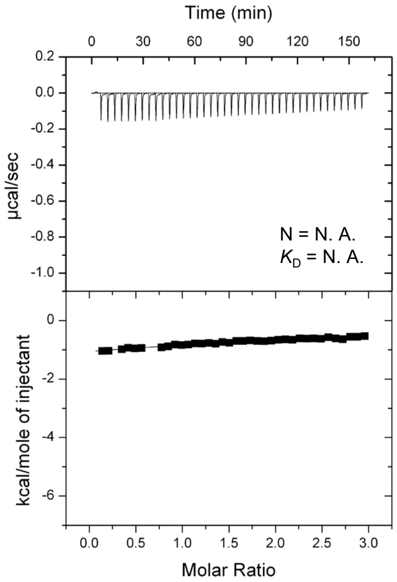

UBR4^{UBR} (F1671A) with REFS

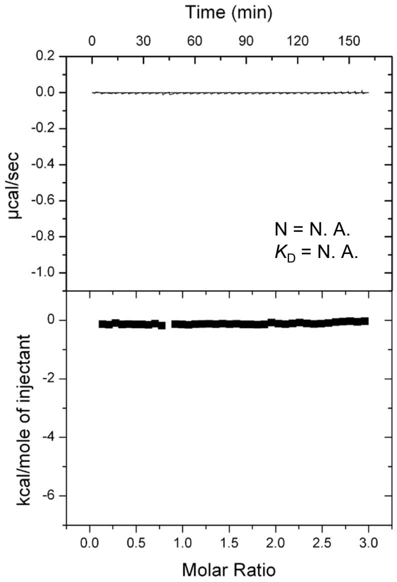

UBR4^{UBR} (F1713A) with YEFS

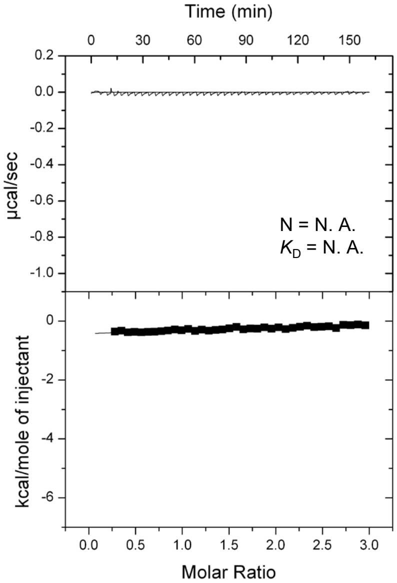

UBR4^{UBR} (F1713A) with REFS

Supplementary Figure 7.

c

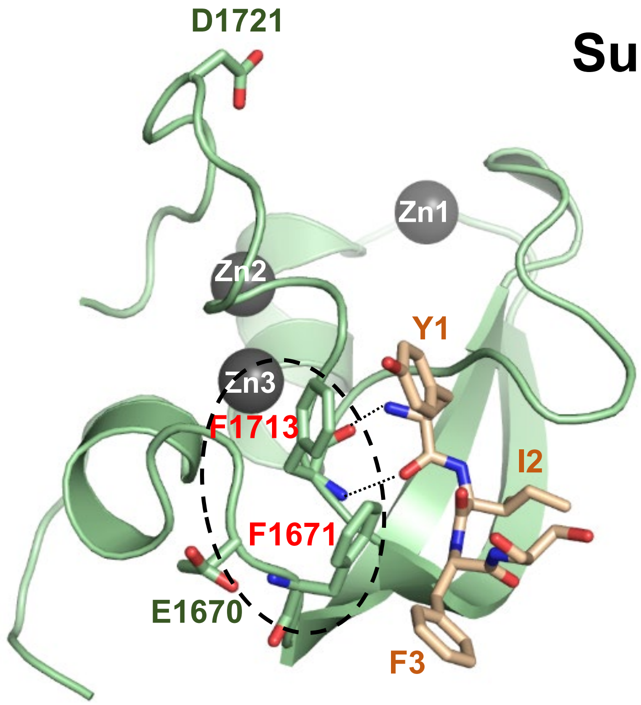

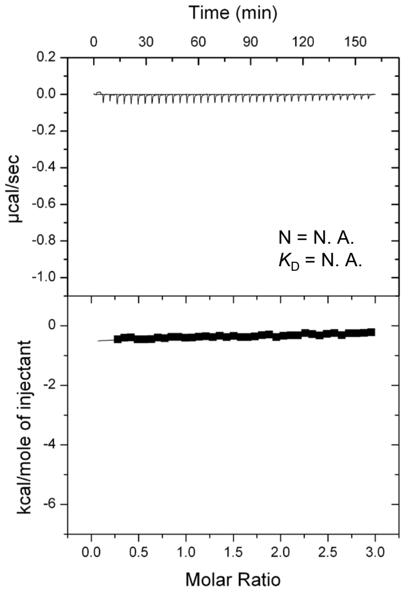

UBR4^{UBR} (F1671I) with YEFS

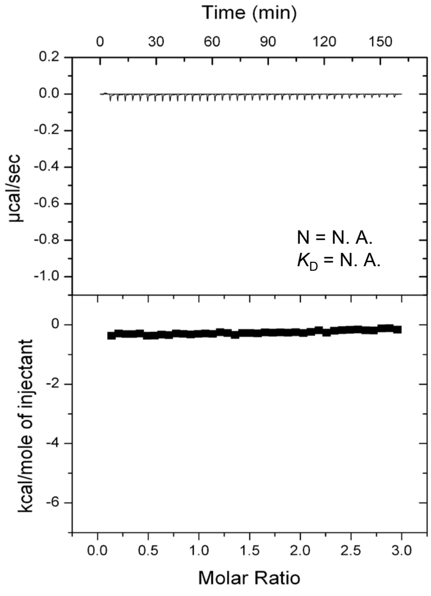

UBR4^{UBR} (F1671I) with REFS

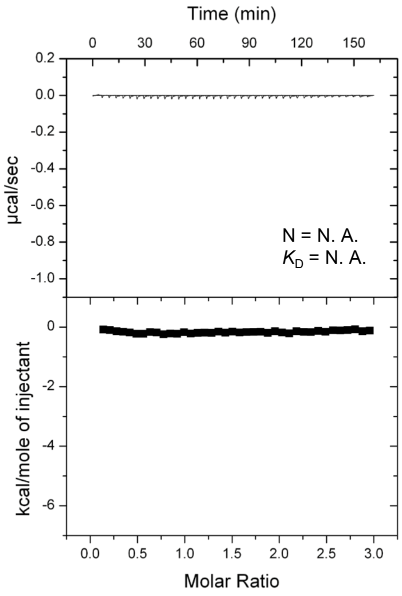

UBR4^{UBR} (F1713I) with YEFS

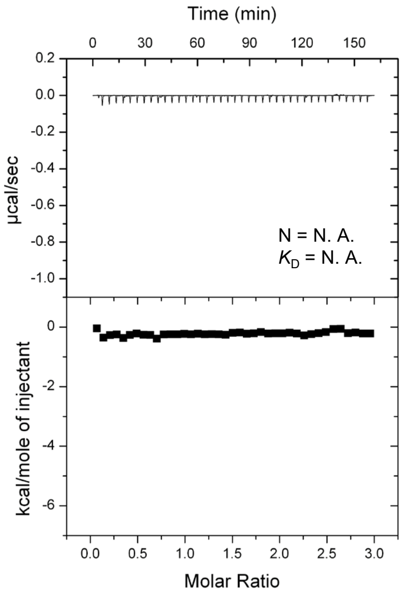

UBR4^{UBR} (F1713I) with REFS

Supplementary Figure 7.

d

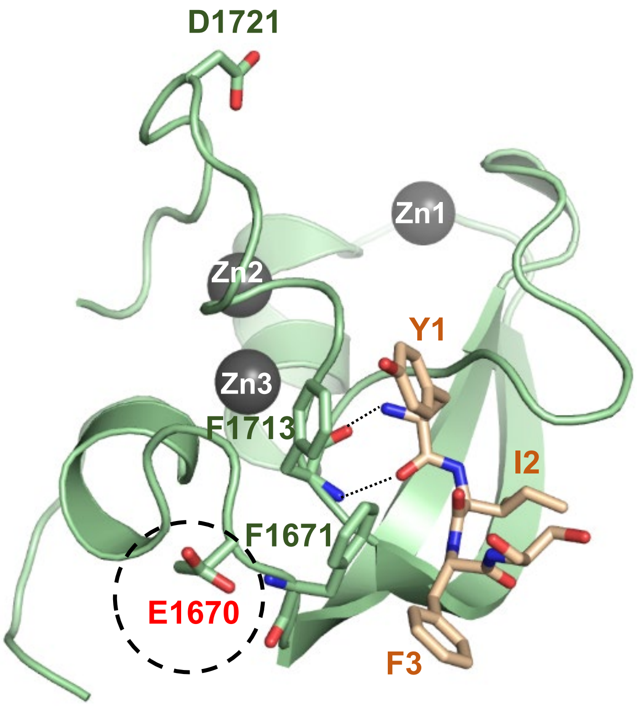

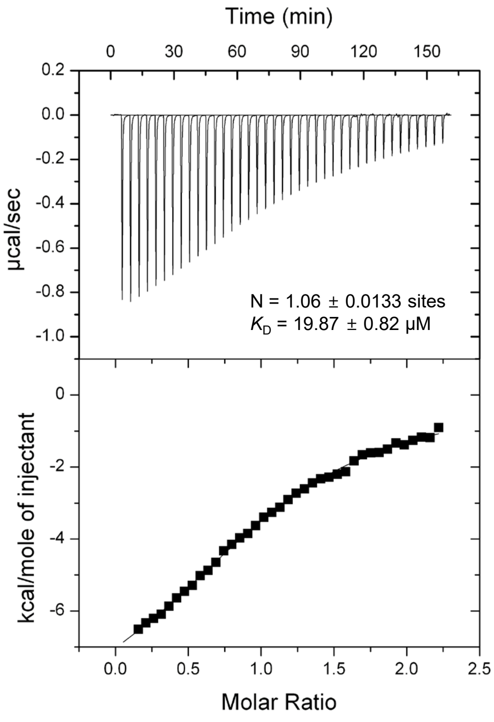

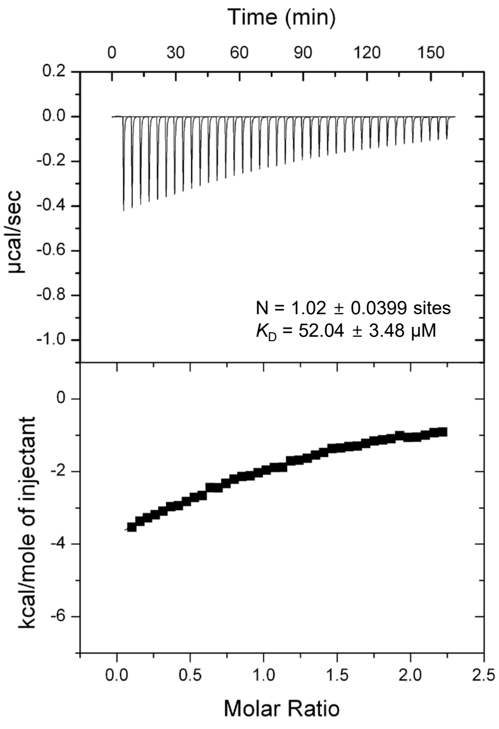

UBR4^{UBR} (E1670A) with YEFS **UBR4^{UBR} (E1670A) with REFS**

Supplementary Figure 7.

Supplementary Fig. 7. (**a**) The D1721 residue, presented using dotted circle, in the YIFS-UBR^{UBR} structure. UBR4^{UBR} (D1721A) with YEFS and UBR4^{UBR} (D1721A) with REFS of raw isothermal titration calorimetry (ITC) data are shown. (**b**) In the crystal structure of YIFS-UBR4^{UBR}, the F1671 and F1713 residues of YIFS-UBR4^{UBR} involved in binding with ligand YIFS are indicated with dotted circles. UBR4^{UBR} (F1671A) with YEFS or REFS and UBR^{UBR} (F1713A) with YEFS or REFS of ITC data shown. (**c**) YIFS-UBR4^{UBR} of F1671 and F1713 residues is depicted with dotted circles. UBR4^{UBR} (F1671I) with YEFS or REFS and UBR^{UBR} (F1713I) with YEFS or REFS of ITC are data shown. (**d**) YIFS-UBR4^{UBR} of E1670 residues is depicted with dotted circles. UBR4^{UBR} (E1670A) with YEFS or REFS of ITC are data shown. The concentrations of each UBR4^{UBR} mutant proteins are used at 0.05 mM and peptides are used at 1 mM.

Supplementary Figure 8.

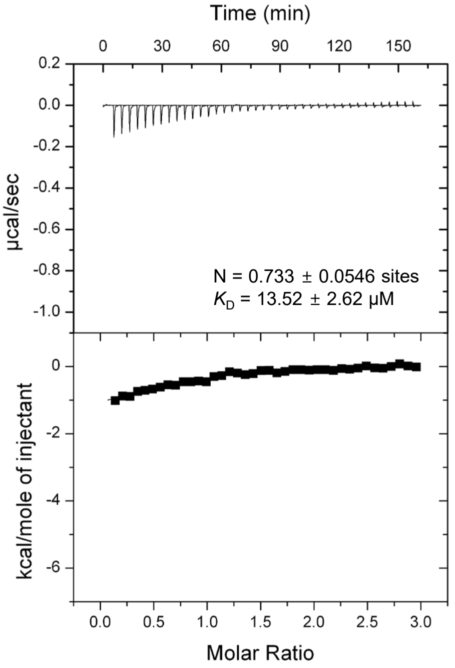

UBR4^{UBR} (K1708A) with YEFS

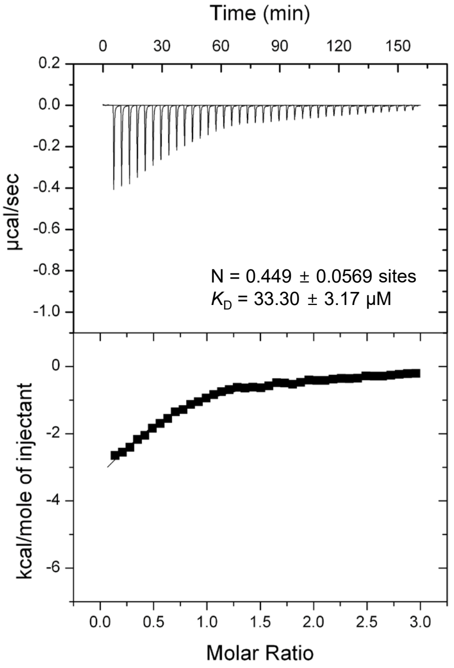

UBR4^{UBR} (K1708A) with YKFS

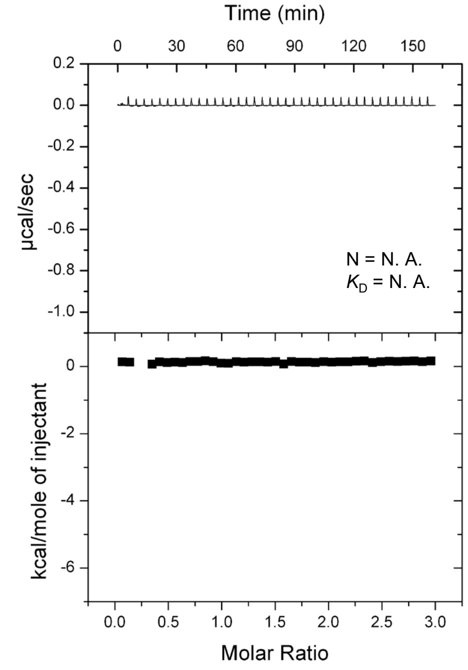

UBR4^{UBR} (K1708A) with YDFS

Supplementary Fig. 8. Raw isothermal titration calorimetry data for the UBR4^{UBR} (K1708A) mutant and three ligands (YEFS, YKFS, and YDFS) of the YXFS peptides. The concentrations of UBR4^{UBR} (K1708A) mutant protein is 0.05 mM and the peptides are 1 mM.
