# Supplementary material for: Insights into the recognition mechanism in the UBR box of UBR4 for its specific substrates
Source: Commun Biol. 2023 Nov 29;6:1214. doi: 10.1038/s42003-023-05602-7 (PMC10687169; doi:10.1038/s42003-023-05602-7)
Supplement: Supplementary file 3 — Description of additional supplementary files [file 42003_2023_5602_MOESM3_ESM.docx]

Description of Additional Supplementary Files

**File name:** Supplementary Data 1

**Description:** The raw data of ITC assay

**File name:** Supplementary Data 2

**Description:** The raw data of TSA assay
